# Study on Vibration Compaction Behavior of Fresh Concrete Mixture with Ternary Aggregate Grading

**DOI:** 10.3390/ma19020259

**Published:** 2026-01-08

**Authors:** Liping He, Fazhang Li, Huidong Qu, Zhenghong Tian, Weihao Shen, Changyue Luo

**Affiliations:** 1 CCCC Fourth Harbor Engineering Co., Ltd., No. 368 Lijiao Road, Haizhu District, Guangzhou 510290, China; 2 College of Water Conservancy and Hydropower Engineering, Hohai University, Nanjing 210098, Chinaluochangyue@126.com (C.L.)

**Keywords:** ternary-graded concrete, vibration energy transfer, dynamic damping, natural frequency, vibration compaction parameters

## Abstract

The vibration compaction behavior of fully graded fresh concrete differs fundamentally from that of conventional two-graded concrete. Based on measured vibration responses of an internal vibrator and sinking-ball tests, an energy transfer model for fully graded concrete was established by incorporating the effects of aggregate-specific surface area, paste–aggregate ratio, dynamic damping, and natural frequency, and the spatiotemporal attenuation of vibration energy in fresh concrete was systematically analyzed. Experimental results indicate that fully graded concrete exhibits a higher energy absorption capacity during the early stage of vibration, with a maximum energy absorption rate of 423 W and a peak energy transfer efficiency of 76.3%, both of which are significantly higher than those of two-graded concrete at the same slump. However, as a dense aggregate skeleton rapidly forms, the energy absorption efficiency of fully graded concrete decreases more rapidly during the middle and later stages of vibration, showing a characteristic pattern of “high initial absorption followed by rapid attenuation.” Through segregation assessment and porosity analysis, a safe vibration energy range for fully graded concrete was quantitatively determined, with lower and upper energy thresholds of 159.7 J·kg^−1^ and 538.5 J·kg^−1^, respectively. In addition, the experiments identified recommended vibration durations of 30–65 s and effective vibration influence radii of 22–85 mm for fully graded concrete under different slump conditions. These findings provide a quantitative basis for the control of vibration parameters and energy-oriented construction of fully graded concrete.

## 1. Introduction

During the vibration compaction of concrete, aggregate gradation and particle shape are among the key factors governing the compaction efficiency of the internal structure [1] and the final compactness [2]. The compaction behavior of concrete during vibration energy transfer and dissipation is essentially a coupled process involving paste filling [3], aggregate sliding [4], and air bubble escape [5], which eventually reaches a stable equilibrium state [6]. Aggregate gradation directly determines the inter-particle void structure [7] and packing density [8], thereby influencing the efficiency of vibration energy transfer within the system [9,10,11]. In traditional hydraulic structures, ternary-graded concrete has been widely used in mass concrete construction due to its relatively low hydration heat [12] and small internal expansion coefficient [1,13].

However, ternary-graded concrete contains a large proportion of coarse aggregates with particle sizes ranging from 40 to 80 mm [14]. Aggregates in this size range are often concentrated in distribution [15]. During spatial arrangement, these large particles tend to form relatively large inter-particle voids [16], which makes it difficult for the paste to fully fill these local voids [17], resulting in great difficulty in achieving complete compaction of ternary-graded fresh concrete mixture [18]. During vibration, the excitation energy of the internal vibrator must first overcome the local friction resistance between large coarse aggregates [19]. As a result, energy dissipation is more concentrated in the contact zones of large particles [20], leading to insufficient paste flowability in some regions [21] and restricted air bubble escape [22]. This easily causes locally uncompacted zones [23]. Because the natural voids between large aggregates are larger [24] and the paste has more difficulty penetrating and filling these voids [25], this type of fresh concrete mixture is generally more difficult to achieve full compaction under the same excitation force [26]. Its overall compaction quality is usually lower than that of concrete with a higher content of fine materials and more uniform particle contact [27].

In addition, the shape of aggregate particles also plays an important role in vibration compaction performance [2,28,29,30]. The large coarse aggregates in ternary-graded concrete are generally angular, multi-faceted particles [31], while the aggregates in binary-graded concrete are mostly reshaped and can be approximated as rounded or elliptical particles [32]. Angular aggregates have rough surfaces and higher friction resistance [33,34], which hinders particle sliding and rearrangement [35]. In contrast, elliptical aggregates can roll, move, and reorient more easily under vibration, which facilitates air bubble escape and structural densification [36,37,38,39]. Therefore, to improve the compactness and mechanical performance of ternary-graded concrete, the vibration parameters used for conventional binary-graded concrete are no longer sufficient. It is necessary to investigate the compaction mechanism and appropriate vibration parameters specifically for ternary-graded fresh concrete mixture.

At present, many valuable achievements have been made in studies on the vibration compaction behavior of concrete. For example, Wang et al. [40] investigated the mechanical properties of concretes with different aggregate gradations and found that aggregate gradation has a significant influence on triaxial compression performance, and concrete mixtures with larger aggregate sizes usually exhibit higher compressive strength. Cook [41] demonstrated that the gradation of fine aggregates affects the cohesion of slipform pavement concrete mixtures, leading to segregation and edge collapse. Li et al. [42] proposed an energy transfer model to evaluate vibration quality, and the results showed that the poorer the flowability of concrete and the higher the aggregate content, the faster the energy attenuation in concrete. However, existing studies mainly focus on the mechanical properties and compaction mechanisms of conventional binary graded concrete. The dynamic structural evolution and compaction mechanism of ternary graded concrete during vibration have not yet been adequately addressed. Therefore, investigating the differences in vibration compaction behavior between ternary graded and binary graded fresh concrete mixtures, and exploring the structural evolution of ternary graded concrete during vibration, is helpful for revealing the relationships among the dynamic damping, natural frequency of aggregates, and the porosity and compactness of hardened concrete.

Existing studies mostly evaluate vibration compaction effectiveness using empirical parameters or macroscopic indices, while energy characterization models established directly from measured vibration responses remain limited. Moreover, a unified quantitative framework describing how the dynamic damping of concrete regulates vibration-induced energy attenuation during compaction is still lacking. Therefore, this study focuses on the mechanisms of energy transfer and dissipation during the vibration compaction of freshly mixed three-graded concrete, with particular attention to the following two key issues—“how the vibration acceleration of the internal vibrator can characterize and map the distribution of energy density within the concrete”; and “how the dynamic damping of concrete governs the spatiotemporal attenuation of vibration energy during the compaction process”. First, based on the “Sinking ball test” [43], the macroscopic vibration compaction behavior of ternary-graded concrete is clarified. An energy transfer model is established by incorporating the flow damping of fresh concrete mixture [44], paste–aggregate ratio [45], specific surface area of coarse aggregates [46], and natural frequency [47], and the energy dissipation law of ternary-graded concrete with increasing distance from the vibration center is analyzed. Second, the roles of aggregate dynamic damping and natural frequency in the absorption of vibration energy during the vibration process of ternary-graded fresh concrete mixture are examined, and the influence of large-size coarse aggregates on the energy absorption efficiency of the mixture is revealed. Finally, based on the energy method [48], a three-dimensional mesoscopic dynamic compaction model of concrete is established to investigate the effect of the volume fraction of large-size coarse aggregates on the overall vibration compaction performance of concrete, and reasonable vibration process control parameters are proposed. This study provides important theoretical significance and practical engineering value for revealing the vibration compaction mechanism of ternary-graded concrete.

## 2. Energy Transfer Model of Ternary-Graded Fresh Concrete Mixture

### 2.1. Energy Transfer Model of Internal Vibrator Based on Grading Factors

**Figure 1:**
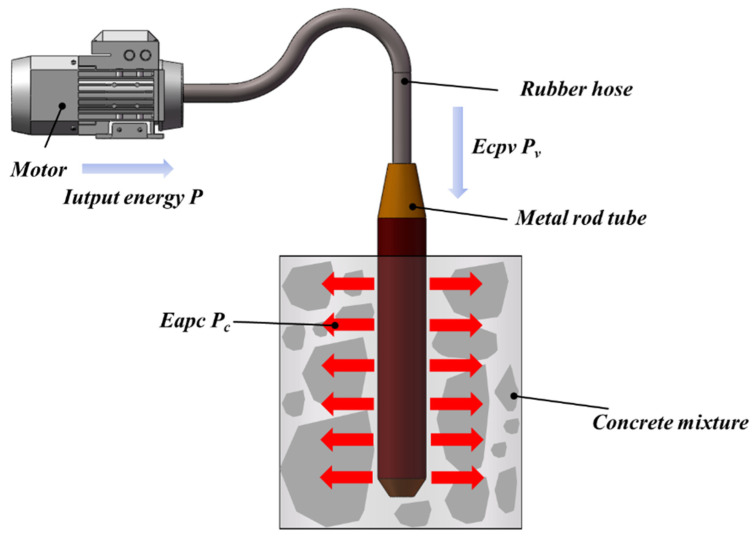
Energy transfer model of vibrating rod.

As shown in Figure 1, when the internal vibrator is inserted into the fresh concrete mixture, part of the motor input power is used to overcome the multiple internal damping effects of the vibrator itself, while the remaining vibration energy propagates outward from the vibrator casing into the surrounding fresh concrete mixture. This process induces relative motion and redistribution of aggregates, mortar, and air bubbles inside the mixture, thereby improving the compactness and uniformity of the concrete [49,50]. Therefore, the input power *P* of the driving motor can be decomposed into the energy absorption power of the fresh concrete mixture, *P*_*c*_ (Energy absorption power of concrete, Eapc), and the energy consumption power of the vibrator, *P_v_* (Energy consumption power of vibrator, Ecpv), as expressed in Equation (1):

(1) $$P=P_{c}+P_{v},$$

where *P* (W) is the input power of the driving motor, *P_c_* (W) is the energy absorption power of the fresh concrete mixture, and *P_v_* (W) is the energy consumption power of the internal vibrator.

#### 2.1.1. Energy Consumption Power of Internal Vibrator

**Figure 2:**
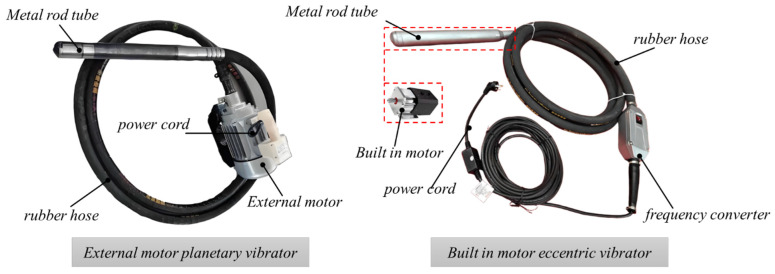
Composition of vibrating rod structure.

As shown in Figure 2, a handheld internal vibrator generally consists of a driving motor, a rubber hose, a metal vibrating tube, and a power cable. The built-in motor is located inside the metal rod tube. According to the installation position of the motor and the vibration driving mode of the metal tube, internal vibrators can be classified into external motor planetary-type vibrators and internal motor eccentric-type vibrators [51]. During operation, the motor converts electrical energy into mechanical energy to drive the eccentric or planetary assembly, causing the metal vibrating tube to generate high-frequency vibration. However, during the transmission of mechanical energy inside the vibrator, part of the energy is dissipated due to friction among the vibrating rod components, structural damping, and vibration absorption of the tube material. As a result, a portion of the input power is continuously converted into sound energy and thermal energy [52]. Therefore, the energy consumption power of the vibrator *P_v_* can be divided into two parts, namely the energy consumption of the vibrator components *P*_1_ and the energy consumption of the vibrator casing *P*_2_, as expressed in Equation (2):

(2) $$P_{v}=P_{1}+P_{2},$$

where *P_v_* (W) is the energy consumption power of the internal vibrator, *P*_1_ (W) is the energy consumption of the vibrator components, and *P*_2_ (W) is the energy consumption of the vibrator casing.

#### 2.1.2. Energy Consumption of Vibrating Rod Assembly

**Figure 3:**
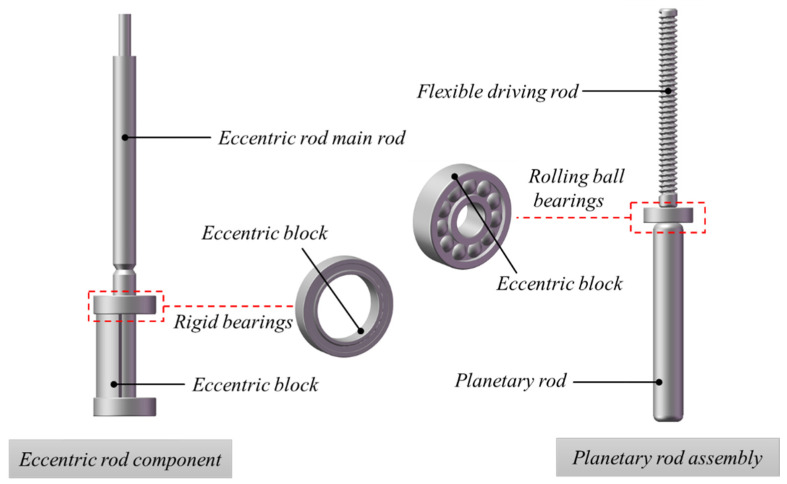
Eccentric Rod/Planetary Rod Component Structure.

As shown in Figure 3, the eccentric rod assembly mainly consists of an eccentric main rod, an eccentric block, and rigid bearings, while the planetary rod assembly is composed of a planetary rod, a flexible driving rod, and self-aligning bearings. The two assemblies are highly similar in structural form, and their core function is to convert the rotational motion generated by the motor into high-frequency vibration. In terms of structural connection, the rods in the assembly are connected to the inner ring of the bearing, while the outer ring of the bearing is fixed to the inner wall of the metal vibrating tube. The rolling elements between the inner and outer bearing rings provide rotational support and force transmission, thereby effectively transferring the excitation force generated by the eccentric or planetary assembly to the metal vibrating tube, enabling it to produce stable and uniform overall vibration [42].

However, under the driving action of the motor, the rod drives the inner ring of the bearing to rotate at a high speed. Relative motion between the inner and outer rings of the bearing is achieved through rolling elements, and during this process, part of the motor input energy is dissipated due to rolling friction and sliding friction [53]. This portion of energy loss can be regarded as the energy consumption of the vibrator assembly, and its power is expressed by Equation (3):

(3) $$P_{1}=M_{f}\cdot \omega ,$$

where *P*_1_ (W) is the energy consumption of the vibrator assembly, *M_f_* (N·m) is the bearing friction torque, and *ω* (rad/s) is the angular velocity of the eccentric or planetary assembly driven by the motor, with *ω =* 2π*n*/60, where *n* (r/min) is the rotational speed of the bearing inner ring.

Based on experimental measurements of bearing friction torque, Rivera [54] pointed out that the friction torque generated during bearing operation is closely related to factors such as angular velocity, bearing load, number of rolling elements, and lubrication conditions. Its magnitude can be calculated using Equation (4):

(4) $$M_{f}=Z\cdot Q\cdot r_{m}\cdot \mu +C_{\eta }\cdot \eta \cdot r_{m}^{3}\cdot \omega ,$$

where *M_f_* (N·m) is the bearing friction torque, *Z* is the number of rolling elements, *Q* (N) is the load carried by a single rolling element, *r_m_* (m) is the average radius of the rolling element or contact point, *μ* is the sliding friction coefficient, *η* (Pa·s) is the dynamic viscosity of the lubricating oil, and *ω* (rad/s) is the angular velocity of the eccentric or planetary assembly driven by the motor, with *ω =* 2π*n*/60, where *n* (r/min) is the rotational speed of the bearing inner ring. *C*_*η*_ is an empirical coefficient related to the bearing geometry and the thickness of the lubrication film.

#### 2.1.3. Energy Consumption of Vibrating Tube of Internal Vibrator

**Figure 4:**
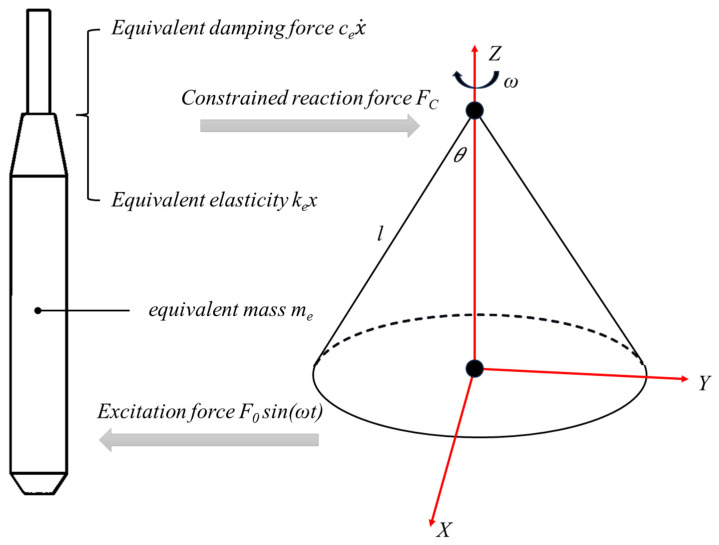
Equivalent dynamic model of vibrating rod tube.

During operation, the metal vibrating tube of the internal vibrator performs a conical pendulum motion under the combined action of the flexible constraint from the rubber hose and the excitation force generated by the eccentric or planetary rod. This motion can be regarded as a uniform spatial rotation of the vibrator tip around a fixed point, forming a conical surface in space, as shown in Figure 4. If the tube length is (*l*) and the swing angle is (*θ*), the spatial displacement trajectory of the vibrator tip can be expressed as

(5) $$\left\{\begin{array}{l} x(t)=lsin\theta cos(\omega t)\\ y(t)=lsin\theta sin(\omega t)\\ z(t)=lcos\theta \end{array}\right..$$

From Equation (5), this motion can be regarded as a spatial conical trajectory of the vibrator tip rotating around a fixed point. In the horizontal plane, it can be equivalently treated as a single-degree-of-freedom harmonic motion perpendicular to the direction of movement [55].

Furthermore, for convenience in dynamic analysis, the flexible constraint provided by the rubber hose can be modeled as a viscoelastic support element with linear stiffness and damping properties. Therefore, under the combined action of the flexible constraint and the periodic excitation force, the equivalent dynamic equation of the vibrating tube can be expressed as

(6) $$m_{e}\ddot{x}+F_{c}=F_{0}\sin(\omega t),$$

(7) $$F_{c}=k_{e}x+c_{e}\dot{x},$$

(8) x=Xsin(ωt−φ),

(9) $$k_{e}=\frac{GA}{L},$$

(10) $$c_{e}=2\xi \sqrt{m_{e}k_{e}},$$

where *F*_0_ (N) is the excitation force generated by the eccentric or planetary rod, *F_c_* (N) is the constraint reaction force, *ω* (rad/s) is the angular velocity of the equivalent model of the metal vibrating tube, *t* (s) is the vibration duration, *m_e_* (kg) is the equivalent mass of the vibrating tube, *k_e_* is the equivalent stiffness of the rubber hose, *c_e_* is the equivalent damping coefficient, *x* (m) is the displacement of the free end of the vibrator relative to the equilibrium position, X is the vibration amplitude at the center of the metal tube, *φ* (rad) is the initial phase of the metal tube, *G* (Pa) is the shear modulus of the rubber, *A* (m^2^) is the cross-sectional area of the hose, L (m) is the effective length of the rubber hose, and ξ is the damping ratio.

Therefore, the energy consumption of the vibrating tube of the internal vibrator per unit time can be expressed as

(11) $$P_{s}(t)=c_{e}{\dot{x}}^{2}(t),$$

(12) $$\dot{x}(t)=\omega Xcos(\omega t-\varphi ),$$

(13) $$P_{2}=\frac{1}{T}\int_{0}^{T}P_{s}(t)dt=\frac{c_{e}{\left(\omega X\right)}^{2}}{2},$$

where $P_{s}(t)$ (W) is the instantaneous energy consumption of the vibrating tube of the internal vibrator, $\dot{x}(t)$ (m/s) is the velocity of the free end of the vibrator relative to the equilibrium position, and $P_{2}$ (W) is the energy consumption of the vibrating tube per unit time.

### 2.2. Energy Diffusion Model in Fresh Concrete Mixture

During vibration compaction, vibrational energy originates from the poker vibrator and propagates into the surrounding concrete medium in the form of mechanical waves. This process induces periodic relative motion among aggregates, mortar, and the pore system within the fresh mixture, thereby overcoming interparticle friction and paste cohesive resistance. As a result, entrapped air is expelled, pores are closed, and particles are rearranged, ultimately leading to progressive densification of the concrete structure. Under conditions where significant vibration-induced segregation of aggregates does not occur, effective transmission of vibrational energy is the key factor governing the quality of vibration compaction. Since aggregate gradation directly determines the internal pore structure, particle contact conditions, and dynamic damping characteristics of concrete, the propagation and attenuation behavior of vibrational energy differ markedly among concretes with different gradations. Therefore, establishing an energy diffusion model that explicitly accounts for the influence of gradation characteristics constitutes an essential theoretical basis for the quantitative evaluation of vibration-induced compaction performance.

#### 2.2.1. Relationship Model Between Acceleration and Energy Density

Vibrational energy in concrete is primarily stored in the form of kinetic energy associated with particle oscillation and elastic strain energy arising from deformation of the mortar–aggregate system. For the convenience of engineering modeling, the instantaneous energy storage state of concrete during vibration is herein equivalently characterized by the energy density per unit volume, denoted as ω, which is assumed to be uniquely determined by the vibration acceleration field. Based on measured vibration response data, and under the assumption that local mesoscopic heterogeneities can be neglected, fresh concrete is treated as an equivalent homogeneous medium. Accordingly, the energy density at an arbitrary location and time can be uniformly expressed as a function of acceleration, as given in Equation (14):

(14) $$\omega =\omega_{k}+\omega_{p},$$

(15) $$\omega_{k}=\frac{1}{2}\rho u^{2}=\frac{1}{2}\rho \cdot \frac{a^{2}}{{\left(2\pi f_{n}\right)}^{2}},$$

(16) $$\omega_{p}=\frac{1}{2}m^{2}\cdot \frac{a^{2}}{{\left(2\pi f_{n}\right)}^{2}},$$

where ω is the energy density per unit volume of concrete, ωₖ is the kinetic energy density, and ωₚ is the potential strain energy density, ρ denotes the density of concrete; u is the vibration displacement induced by the poker vibrator, a is the vibration acceleration of the concrete, f_n_ is the natural vibration frequency of the concrete, and m is the mass of concrete per unit volume.

#### 2.2.2. Regulation Mechanism of Energy Attenuation by Dynamic Damping

Based on viscous damping wave theory, the attenuation of energy density along the radial direction *r* follows an exponential decay model. The concrete dynamic damping coefficient λₘ governs the radial attenuation characteristics of vibrational energy, such that the energy density decreases exponentially with increasing distance. Accordingly, the spatial distribution of energy density can be expressed as

(17) $$\omega =\omega_{0}\cdot \exp\left(-\lambda_{m}\cdot r\right),$$

where ω_0_ is the initial energy density at the boundary of the poker vibrator, *r* is the radial distance from the vibration source, and λₘ is the dynamic damping coefficient of the concrete.

#### 2.2.3. Coupling Effect of Coarse Aggregate-Specific Surface Area and Natural Frequency

According to the particle-size ranges of the three-graded coarse aggregates (5–20 mm, 20–40 mm, and 40–80 mm), the median particle diameters *d*ᵢ of each range are taken as 12.5 mm, 30 mm, and 60 mm, respectively. Assuming the aggregates to be spherical, the specific surface area *S*ₐ is calculated by mass-weighted averaging as follows:

(18) $$S_{i}=4\pi r_{i}^{2},$$

(19) $$S_{a}=\sum_{i=1}^{3}\frac{m_{a,i}\cdot S_{i}}{m_{a,t}},$$

where S_i_ is the surface area of a single coarse-aggregate particle, r_i_ is the radius of a single coarse-aggregate particle, S_a_ is the specific surface area of coarse aggregate in concrete, m_a,i_ is the mass of coarse aggregate within a given particle-size interval, m_a,t_ is the total mass of concrete, and S_i_ denotes the surface area of an individual particle.

The natural frequency of pure mortar, f_0_, decreases with increasing S_a_. A larger S_a_ corresponds to a greater contact area between aggregate and mortar, which enhances interfacial energy dissipation and reduces the equivalent stiffness, thereby leading to attenuation of f_n_. Accordingly, the calculation model for the coarse aggregate-specific surface area S_a_ is given as follows:

(20) $$f_{n}=f_{0}\left(1-\alpha S_{a}\right),$$

where α is the influence coefficient of specific surface area; f_n_ is the natural frequency of concrete; f_0_ is the natural frequency of pure mortar; and S_a_ is the specific surface area of coarse aggregate in concrete.

As the specific surface area S_a_ of coarse aggregate increases, the interfacial viscous friction energy dissipation between aggregate and mortar becomes more pronounced. Consequently, the energy attenuation per unit distance, Δω, exhibits a linear positive correlation with S_a_. By introducing the contact energy dissipation coefficient β, the attenuation term is augmented accordingly:

(21) $$\Delta \omega =\beta S_{a}+\left(1+0.5\varepsilon \right)\varepsilon ,$$

where Δ*ω* is the energy attenuation per unit distance, *β* is the contact energy dissipation coefficient, *S*_*a*_ is the specific surface area of coarse aggregate in concrete, and *ε* is the inter-aggregate pore porosity.

#### 2.2.4. Energy Diffusion Model

Based on the above relationships and in combination with the harmonic vibration characteristics of spherical waves, the energy density distribution E_i_ at an arbitrary radial distance r and time t is finally obtained. Furthermore, by integrating the energy density over the vibration duration t and within the effective influence radius R, the cumulative absorbed energy per unit volume of concrete, E_t_, can be determined:

(22) $$E_{i}=e_{0}e^{-\Delta \omega r}\cdot \sin^{2}\left(2\pi f_{n}t\right),$$

(23) $$E_{t}=\int_{0}^{t_{0}}\int_{r_{0}}^{R}e_{0}e^{-\Delta \omega r}\cdot \sin^{2}\left(2\pi f_{n}t\right)\cdot 4\pi r^{2}drdt,$$

where *e*_0_ is the initial energy density at the boundary of the vibrator casing (*r* = *r*_0_), *r* is the radial distance from the center of the poker vibrator, *t* is the vibration duration, *R* is the effective vibration influence radius, and *f*_*n*_ is the natural frequency of the concrete.

It should be noted that the equations presented in Section 2.1 and Section 2.2 are derived or adapted from existing vibration and energy-transfer theories, and their role is to provide a physically consistent framework for interpreting acceleration-based measurements, rather than serving as independent empirical fitting models.

## 3. Experimental Program and Case Study

### 3.1. Experimental Materials and Equipment

The cement used in this study was ordinary Portland cement, conforming to GB 175-2023 [56], with a density of 3020 kg/m^3^. Fine aggregates consisted of a blend of natural river sand and manufactured sand, mixed at a mass ratio of 6:4. The particle size distribution, apparent density, and clay content of the fine aggregates were determined in accordance with GB/T 14684–2022 [57], and the apparent density of the blended fine aggregate was 2540 kg/m^3^. Coarse aggregates were granite crushed stone with particle size ranges of 5–20 mm, 20–40 mm, and 40–80 mm, forming a fully graded aggregate system. The particle gradation, flakiness index, crushing value, and apparent density of the coarse aggregates were tested according to GB/T 14685–2022 [58], and the apparent density was 2620 kg/m^3^.

The mineral admixtures included Class I fly ash and S95-grade ground granulated blast-furnace slag (GGBS), which were incorporated at a mass ratio of 5:5. The fly ash satisfied the requirements of GB/T 1596–2017 [59], while the GGBS met the specifications of GB/T 18046–2017 [60]. A corrosion-resistant enhancing agent (570P-HGCPA, Shandong Landu New Materials Co., Ltd., Shandong, China) was used, and tap water from the laboratory was employed as the mixing water. A high-performance polycarboxylate-based superplasticizer was adopted to regulate the workability of the mixtures.

The concrete mix proportions were designed in accordance with JGJ 55–2011 [61]. Under the premise of satisfying the target slump and workability requirements, both two-graded and fully graded fresh concrete mixtures with different flow-induced damping characteristics were prepared by adjusting the water-to-binder ratio and aggregate gradation.

All internal vibrators used in the experiments were of the built-in motor and eccentric rod type. The basic parameters of the internal vibrator in air are listed in Table 1. The bearing friction coefficient μ was adopted from typical values reported for grease-lubricated rolling bearings under high-speed operating conditions, and variations within the common range (0.001–0.002) were found to have a negligible influence on the calculated vibration energy distribution.

### 3.2. Experimental Design and Procedure

To compare the vibration compaction behavior of ternary-graded concrete and binary-graded concrete, two experiments were designed in this study. Experiment I was conducted to investigate the effective vibration radius and flow damping of ternary-graded fresh concrete mixture. The fresh ternary-graded concrete mixture was poured into a concrete mold with dimensions of 80 cm in length, 50 cm in width, and 40 cm in height. Colored glass beads were placed on the top surface as tracer particles. Meanwhile, acceleration sensors were embedded at distances of 10 cm, 20 cm, 30 cm, and 40 cm from the vibration center to measure the actual acceleration of the fresh concrete mixture at different locations.

**Figure 5:**
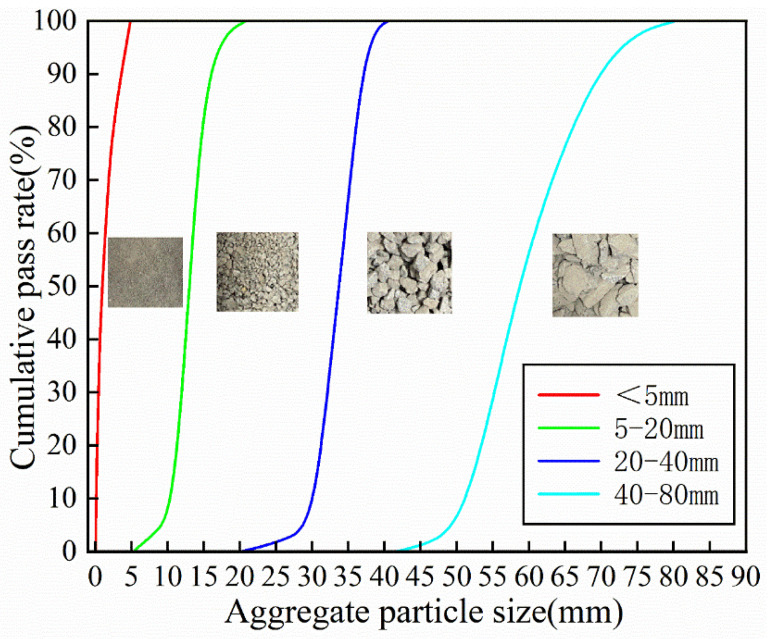
Distribution of aggregate particle size distribution in concrete samples.

The internal vibrator was inserted at the leftmost side of the mold for vibration. After vibration was completed, measurements were taken with the center of the vibrator as the reference point. When 90 percent of the glass beads had settled into the fresh concrete mixture, it was taken as the detection criterion, and the vibration influence radius of the ternary-graded concrete was determined. The particle size distribution of aggregates in the concrete specimens is shown in Figure 5.

The output power of the motor was recorded using a power analyzer (Beijing Oriental Institute of Vibration and Noise Technology, Beijing, China) connected to the motor. The acceleration and vibration frequency of the vibrator casing were obtained using an acceleration acquisition system consisting of a signal analyzer and accelerometers. The insertion depth of the internal vibrator was specified as 35 cm. Therefore, the midpoint, located 17.5 cm from the top of the casing head, was selected as the representative position, where the accelerometer was installed.

**Figure 6:**
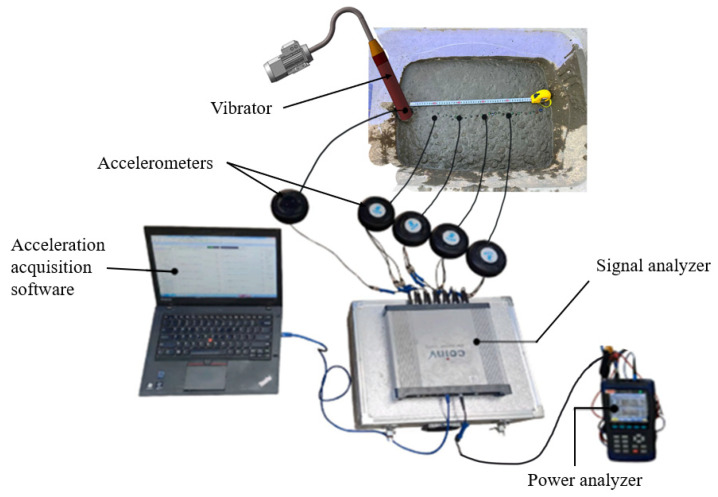
Connection of parameter acquisition equipment in Experiment 1.

The vibration parameters were collected using an INV3062A4 signal acquisition and analysis system (Beijing Oriental Institute of Vibration and Noise Technology, Beijing, China) and B02-series triaxial accelerometers (Beijing Oriental Institute of Vibration and Noise Technology, Beijing, China), in which the B02B72 sensor (Beijing Oriental Institute of Vibration and Noise Technology, Beijing, China) was used at the center of the internal vibrator and the B02Y70 sensor (Beijing Oriental Institute of Vibration and Noise Technology, Beijing, China) was embedded in the fresh concrete mixture. By synchronously acquiring acceleration signals at different positions, the spatial attenuation law of vibration energy was obtained. The equipment connection setup is shown in Figure 6.

The material damping coefficient *λ* and the influence radius R of the internal vibrator were determined through experiments using the connection arrangement shown in Figure 6. Accelerometers were embedded at distances of 10 cm, 20 cm, 30 cm, and 40 cm from the vibration source. The acceleration data of both the vibrator casing and the fresh concrete mixture were collected, and the material damping coefficient of different concrete mixtures was obtained by fitting the data using Equation (24):

(24) $$a_{l}=a\xi_{b}\frac{1}{\sqrt{l}}e^{-\xi ml}.$$

To determine the material damping coefficient (λ) and the vibration influence radius (R), acceleration responses were measured at four radial distances (10, 20, 30, and 40 cm) from the vibrator axis using embedded accelerometers. For each test, the steady-state root mean square (RMS) acceleration amplitude at each location was extracted from the time histories after eliminating the initial transient stage.

According to Equation (24), the spatial attenuation of vibration energy was assumed to follow an exponential decay form. The measured acceleration amplitudes were first converted into corresponding energy density values and then fitted to Equation (24) as a function of radial distance. A nonlinear least-squares regression was performed to identify the material damping coefficient λ, while the influence radius R was defined as the distance at which the fitted energy density decayed to 10% of its initial value at the vibrator boundary.

The fitting procedure was implemented using MATLAB-2024 with the built-in nonlinear regression function. The goodness of fit was evaluated using the coefficient of determination (R^2^). For all tested mixtures, the fitted results showed good agreement with the experimental data, with R^2^ values ranging from 0.92 to 0.97, indicating that Equation (24) adequately describes the spatial attenuation behavior of vibration energy in fresh concrete.

In the experiments, the settling ball method and the acceleration method were jointly used to characterize the damping properties. The vibration duration was controlled within 60–120 s, and the sampling frequency was set to 4000 Hz. The acceleration signals were integrated to obtain the velocity and displacement response curves, which were used to analyze the processes of concrete liquefaction, air bubble escape, and structural densification.

**Figure 7:**
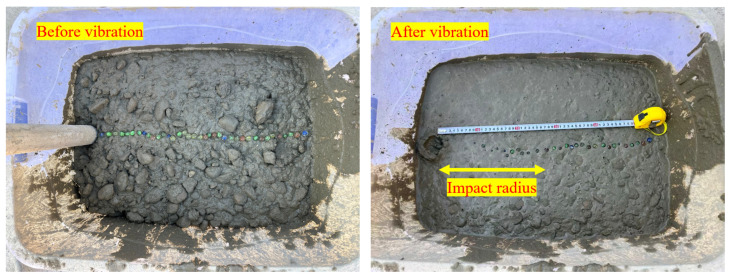
Test for Measuring Material Damping Coefficient.

Glass beads were arranged in a straight line and placed on the surface of the fresh concrete mixture as tracer particles. The internal vibrator continued vibrating until the surface tracer particles no longer sank, after which the vibration was stopped. The particle closest to the vibrator and the vibration source was taken as the influence radius of the vibrator. Figure 7 shows the experimental setup for measuring the vibration influence radius and damping coefficient of ternary-graded fresh concrete mixture.

Experiment II was conducted to investigate the differences in the influencing factors of energy absorption between ternary-graded concrete and binary-graded concrete. The considered factors included insertion depth, natural frequency, dynamic damping, specific surface area and paste–aggregate ratio, as well as interfacial friction. Based on the experimental results, quantitative models of these influencing factors for ternary-graded fresh concrete mixture were established. A total of 10 groups of binary-graded and ternary-graded concrete specimens were designed and cast into circular concrete molds with a diameter of 100 cm and a height of 50 cm. An internal vibrator with a diameter of 70 mm was then inserted for vibration. Meanwhile, acceleration sensors were embedded at distances of 10 cm, 20 cm, 30 cm, and 40 cm from the vibration center to measure the actual acceleration of the fresh concrete mixture, which was used to calculate the actual energy absorption at these four positions.

The rheological properties of concrete are closely related to its flowability. As the flowability increases, the damping of concrete decreases. To investigate the influence of flowability on the energy absorption and distribution of ternary-graded fresh concrete mixture, as well as the difference in flow damping between ternary-graded and binary-graded concrete, a total of 10 groups of concrete specimens, labeled C1–C5 and D1–D5, were prepared for comparative analysis, as is followed in Table 2.

Concrete was cast into a cylindrical wooden mold with a diameter of 100 cm and a height of 50 cm. Acceleration sensors were embedded at distances of 10 cm, 20 cm, 30 cm, and 40 cm from the vibration center to measure the actual acceleration of the fresh concrete mixture, which was used to calculate the energy absorbed at these four positions.

First, the fresh concrete mixture was poured to a height of 15 cm in the mold, and the internal vibrator was inserted to a depth of 10 cm for vibration while the peripheral acceleration was recorded. Then, the vibrated concrete was removed, and new fresh concrete mixture was added to a height of 25 cm. The vibrator was inserted to a depth of 20 cm for vibration, and the peripheral acceleration was recorded again. Next, the vibrated concrete was removed and replaced with fresh concrete mixture to a height of 35 cm. The vibrator was inserted to a depth of 30 cm for vibration, and the peripheral acceleration was recorded. Finally, the vibrated concrete was removed, and new fresh concrete mixture was added to a height of 45 cm. The vibrator was inserted to a depth of 40 cm for vibration, and the peripheral acceleration was recorded.

**Figure 8:**
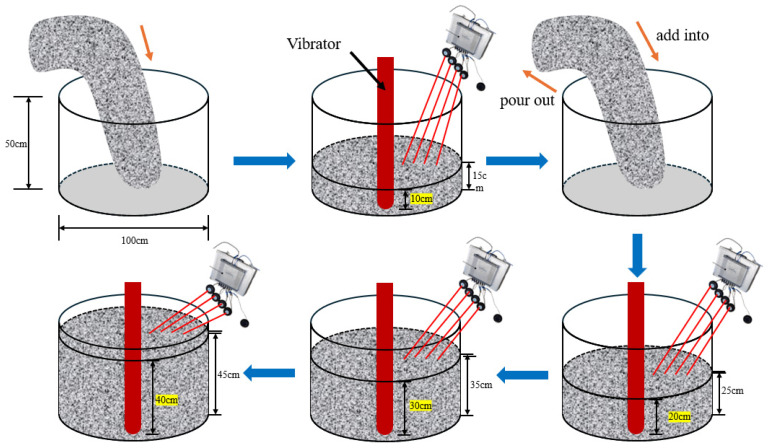
Schematic Diagram of Experiment 2 Process.

Based on the above parameters and the energy transfer model, the energy distribution within the fresh concrete mixture was quantified. After 28 days of curing, concrete specimens from different positions were tested and analyzed, and the energy threshold for effective compaction was determined according to the energy distribution. To ensure the accuracy of the experimental results, each test group was repeated to avoid significant errors and to improve the reliability of the results. A schematic diagram of the experimental procedure is shown in Figure 8.

## 4. Experimental Results and Discussion

### 4.1. Effects of Flow Damping and Aggregate Proportion on Energy Transfer in Ternary-Graded Concrete

#### 4.1.1. Comparison of Effects of Dynamic Damping on Energy Transfer in Binary-Graded and Ternary-Graded Fresh Concrete Mixture

In this study, the term energy absorption capacity of concrete refers to the instantaneous power absorbed by the fresh concrete, denoted as $P_{c}$ (W), rather than cumulative energy. It represents the rate at which vibration energy transmitted from the vibrator is dissipated within the concrete mixture at a given time. To avoid ambiguity, cumulative absorbed energy is not discussed in this section. All energy-related quantities are expressed in terms of instantaneous power unless otherwise specified. Furthermore, the energy utilization rate is defined as the ratio between the instantaneous power absorbed by the concrete and the electrical input power of the vibrator motor, which can be expressed as

$$\eta =\frac{P_{c}}{P_{\mathrm{in}}}\times 100\%,$$

where $P_{c},$ is the instantaneous energy absorption power of the concrete and $P_{\mathrm{in}}$ is the motor input power measured during vibration.

The damping characteristics of concrete are closely related to its flowability and aggregate gradation, and these damping properties further affect the energy absorption efficiency of the internal vibrator. A larger slump indicates better flowability, which corresponds to smaller damping of the fresh concrete mixture. Under this condition, the casing acceleration of the internal vibrator becomes higher, leading to increased power consumption of the vibrator due to its internal friction. More vibration energy is therefore dissipated as internal heat of the vibrator, and correspondingly, less energy is absorbed by the fresh concrete mixture. As a result, the energy utilization efficiency of the concrete with respect to the vibrator is reduced.

**Figure 9:**
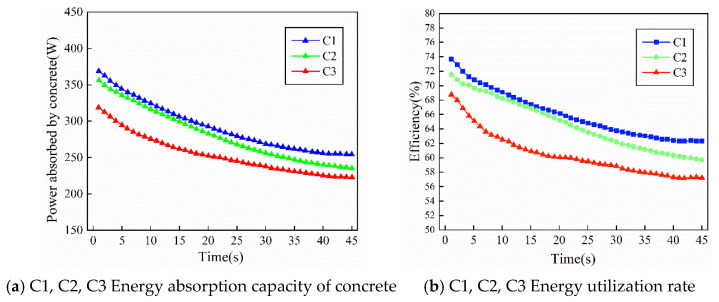
Energy absorption response of second graded concrete under different flow damping.

Figure 9a,b show the variations in energy absorption and energy utilization efficiency with time for binary-graded fresh concrete mixtures with different slumps under the same gradation, corresponding to specimens C1, C2, and C3, respectively. As shown in the figures, for binary-graded concrete, the maximum energy absorption per unit time can reach 374 W at the beginning, and the maximum energy utilization efficiency can reach 74 percent. With the increase in vibration time, the energy absorbed from vibration gradually decreases, and the energy utilization efficiency also shows a decreasing trend. This is because a large number of voids exist inside the concrete at the initial stage, and the coarse aggregates are irregularly stacked. The initial vibration energy is mainly used to overcome the sliding friction of coarse aggregates, promote their dense rearrangement, and disturb the coarse aggregates to prevent interlocking and the formation of closed voids. As vibration continues, the coarse aggregates gradually disperse and settle, and the voids between aggregates are significantly reduced. Subsequently, the vibration energy is mainly used for disturbing the fine aggregates and cement paste to expel air bubbles from the colloidal system. Since this process requires less energy, both the energy absorption and the energy utilization efficiency decrease accordingly with increasing dynamic damping.

**Figure 10:**
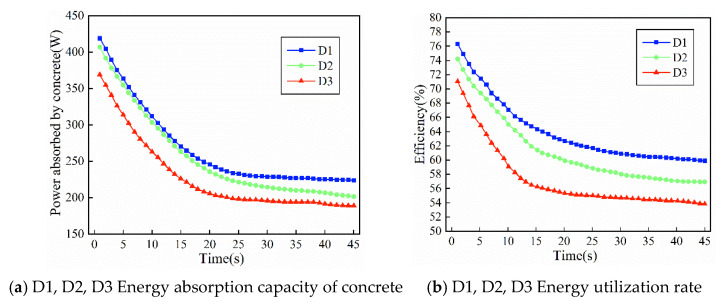
Energy absorption response of three graded concrete under different flow damping.

Figure 10a,b show the variations in energy absorption and energy utilization efficiency with time for ternary-graded fresh concrete mixtures with different slumps under the same gradation, corresponding to specimens D1, D2, and D3, respectively. As shown in the figures, the influence of dynamic damping on the energy utilization of ternary-graded fresh concrete mixture follows a similar trend to that of binary-graded concrete. With increasing slump and decreasing dynamic damping, both the energy absorption and the energy utilization efficiency decrease. In the initial stage, when the dynamic damping of the fresh concrete mixture is relatively large, the energy absorption and utilization efficiency with respect to the internal vibrator are both high. The maximum energy utilization per unit time can reach 423 W, and the highest energy utilization efficiency can reach 76.3 percent.

However, by comprehensively comparing Figure 9 and Figure 10, it can be seen that the influence of dynamic damping on energy absorption in ternary-graded concrete is different from that in binary-graded concrete. With increasing time, the energy absorption efficiency of binary-graded concrete shows a slow decreasing trend. This is because relatively large structural voids exist between coarse aggregates in the binary-graded system, and the number of particle contact points is limited. During the middle and later stages of vibration, a certain amount of rearrangement space is still preserved, allowing part of the vibration energy to continue being converted into effective work. As a result, a relatively low but stable level of energy absorption is maintained. In contrast, ternary-graded concrete can form a stable skeleton at an early stage of vibration. The mutual filling effect among particles leads to rapid compression of voids, and the compactness of the concrete quickly approaches its theoretical limit within a short time. Therefore, although the ternary-graded system exhibits a significantly higher energy absorption efficiency than the binary-graded system at the initial stage of vibration, the rapid locking of the skeleton prevents further particle rearrangement, causing a much faster decline in energy absorption efficiency at the later stage.

From an energy perspective, the effective energy absorption of concrete during vibration mainly includes two parts: the mechanical work required for particle rearrangement and the energy consumed during void closure and air bubble escape. After these processes are completed in ternary-graded concrete, the input vibration energy is no longer effectively absorbed. Instead, a larger proportion of the energy is dissipated as heat through inter-particle friction or is directly transmitted to the surrounding medium. This results in a lower energy absorption efficiency in the later stage compared with binary-graded concrete of the same gradation. Correspondingly, although the casing acceleration and external input power measured by the vibration system remain relatively high at this stage, they no longer produce an obvious effect on the concrete structure itself.

Therefore, the two types of gradation exhibit distinct energy absorption characteristics. Binary-graded concrete shows insufficient energy absorption at the early stage and a slow attenuation at the later stage, whereas ternary-graded concrete is characterized by rapid energy absorption at the early stage and rapid attenuation at the later stage. This difference reflects the fundamental distinctions between the two gradation systems in terms of particle contact state, evolution of spatial degrees of freedom, and the growth rate of damping. Due to the rapid densification of its structure, ternary-graded concrete quickly enters a stage of “vibration without further densification” after reaching the target compactness. In contrast, binary-graded concrete, owing to its loose pore structure and delayed skeleton formation, exhibits a relatively smooth energy absorption curve throughout the entire vibration process.

#### 4.1.2. Comparison of Effects of Aggregate Gradation on Energy Transfer in Binary-Graded and Ternary-Graded Fresh Concrete Mixtures

**Figure 11:**
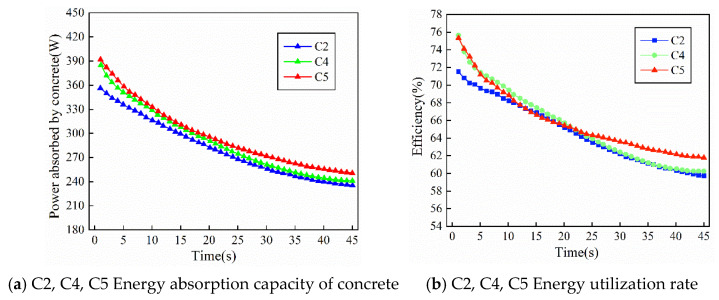
Energy absorption response of second grade concrete under different gradations.

Concrete with different aggregate proportions can also significantly affect the vibration state of the internal vibrator and the ability of the concrete to absorb external energy. Figure 11a,b show the variations in energy absorption and energy utilization efficiency with time for binary-graded fresh concrete mixtures with different gradations under the same slump, corresponding to specimens C2, C4, and C5, respectively. As shown in the figures, among the binary-graded concretes with different particle size distributions, when the proportion of 5–20 mm aggregates increases and the proportion of 20–40 mm aggregates decreases correspondingly in specimen C5, the energy utilization efficiency of C5 becomes higher than that of the standard specimen C2, and it reaches the optimal vibration energy absorption for maximum compactness more rapidly. When the proportion of 5–20 mm aggregates decreases and the proportion of 20–40 mm aggregates increases correspondingly in specimen C4, the energy absorption at the early stage of vibration is also higher than that of the standard specimen C2. However, in the later stage after the concrete becomes compacted, the energy utilization efficiency of specimen C4 becomes close to and gradually converges with that of specimen C2.

**Figure 12:**
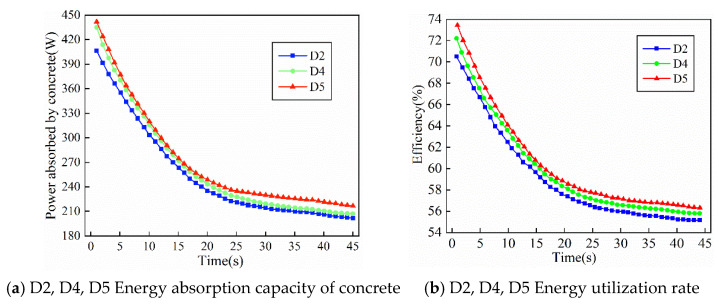
Energy absorption response of three graded concrete with different grades.

Figure 12a,b show the variations in energy absorption and energy utilization efficiency with time for ternary-graded fresh concrete mixtures with different gradations under the same slump, corresponding to specimens D2, D4, and D5, respectively. As shown in the figures, for ternary-graded concrete with different particle size distributions, the energy transfer efficiency generally shows a decreasing trend with time, but it remains above 55 percent throughout the entire vibration process. When the slump is smaller and the aggregate proportion is higher, the concrete system exhibits larger damping, the energy conversion during vibration becomes more sufficient, and thus the energy transfer efficiency is higher, with the average efficiency reaching a maximum of 73.5 percent.

With an increase in the proportion of 5–20 mm aggregates and a corresponding decrease in the proportion of 40–80 mm aggregates, the energy utilization efficiency of specimen D5 is higher than that of specimen D4, and the energy utilization efficiency of specimen D4 is higher than that of the standard specimen D2. Moreover, these specimens reach the optimal vibration energy absorption and utilization for maximum compactness more rapidly. This indicates that, in ternary-graded concrete, when the size of coarse aggregates is gradually reduced, the internal voids can be filled more rapidly, allowing the concrete to reach a dense state more quickly.

This is because changes in coarse aggregate gradation not only modify the compact internal structure of concrete but also directly affect the natural frequency and equivalent damping characteristics of the concrete system. When the proportion of medium-sized aggregates in the range of 5–20 mm increases, the interlocking and filling effects among aggregates become more pronounced. As a result, the porosity of the concrete is significantly reduced, and the overall density is increased. Under this condition, the reaction force acting on the internal vibrator within the concrete becomes larger, which is equivalent to an increase in the working load of the vibrator, and this is manifested as a reduction in casing acceleration. However, in order to overcome the increased resistance, the motor must output more power. This additional mechanical energy input is transferred to a greater extent into the fresh concrete mixture, thereby increasing the power absorbed by the concrete.

From the time evolution of the vibration process, the power absorbed by the fresh concrete mixture and the total power consumed by the internal vibrator exhibit opposite trends. With increasing vibration time, the energy absorption capacity of the concrete gradually decreases, whereas the input power of the vibrator increases slowly and eventually reaches a stable state. This phenomenon can be explained by the evolution of the contact condition between the fresh concrete mixture and the vibrator casing. When the vibrator is first inserted into the fresh concrete mixture, the fluid concrete tightly wraps around the casing, leading to the maximum contact area and the highest boundary damping. As a result, the casing acceleration is at its lowest, while the effective excitation acceleration acting on the concrete is at its highest. At this initial stage, the externally input energy is mainly used for particle rearrangement, void closure, and the upward escape of air bubbles, so the energy absorption power of the concrete reaches its maximum level. However, as vibration continues, the concrete surrounding the casing rapidly expands and becomes dense due to particle rearrangement, forming a relatively stable “local skeleton layer” around the casing. The degree of freedom of this layer decreases, which reduces the contact tightness between the concrete and the vibrator and lowers the boundary damping. Consequently, the casing acceleration increases. Meanwhile, the rearrangeable space within the concrete gradually disappears, the effective work that can be absorbed is significantly reduced, and the energy absorption power of the concrete decreases monotonically with time and eventually becomes stable.

Because ternary-graded concrete has a continuous aggregate gradation and sufficient particle interlocking, fine aggregates can effectively fill the gaps between large aggregates, enabling the concrete to rapidly reach a high degree of compactness at the early stage of vibration. The ternary-graded system is therefore able to absorb a large amount of energy at the early stage, with a higher energy conversion efficiency than that of binary-graded concrete. However, precisely because of its rapid densification, the “local skeleton layer” around the casing forms within a short time, leading to a sharp reduction in the number of free particles, a decrease in damping, and a rapid decline in the energy absorption capacity at the middle and later stages. This is the fundamental reason why ternary-graded concrete exhibits the typical characteristic of high energy absorption at the early stage and rapid attenuation at the later stage.

### 4.2. Energy Dissipation of Ternary-Graded Concrete with Vibration Depth

#### Effect of Vibration Depth on Energy Transfer in Ternary-Graded Fresh Concrete Mixture

The impedance of the ternary-graded fresh concrete mixture varies dynamically under vibration, which makes the transmission of vibration energy time-dependent. With increasing vibration duration, the energy consumption of the mixture per unit time gradually decreases, while the energy consumption of the internal vibrator gradually increases, and both finally tend to stabilize. The reason is that when the internal vibrator is first inserted into the fresh concrete mixture, the mixture tightly wraps the vibrator casing, which forcibly reduces the vibration intensity of the casing. At this stage, the mixture mainly exhibits a solid-like state with a relatively large boundary damping coefficient, which is favorable for the transmission of energy waves, so that a relatively large amount of effective energy acts within the mixture. As the vibration duration increases, the mixture around the casing rapidly becomes liquefied, and a stable liquid paste film gradually forms near the casing. This reduces the contact tightness between the mixture and the casing, thereby increasing the vibration intensity of the casing. However, the paste film lowers the boundary damping coefficient and hinders the transmission of energy waves, resulting in a gradual weakening of the vibration state of the mixture.

**Figure 13:**
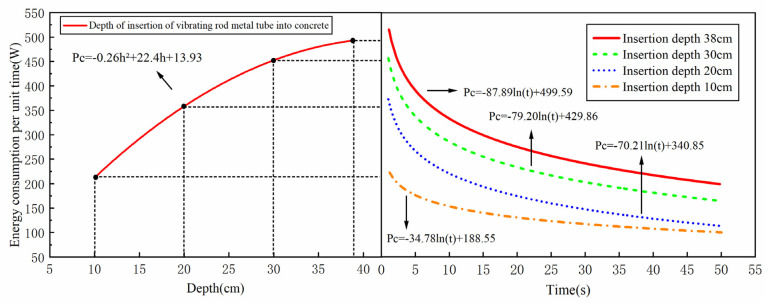
Relationship between energy consumption of three graded concrete mixture and insertion depth of vibrating rod.

When the internal vibrator is inserted into the same fresh concrete mixture at different depths, the influence of mixture impedance shows clear differences. As shown in Figure 13, taking the ZDNE50 built-in motor eccentric-type internal vibrator inserted into concrete specimen D2 as an example, with increasing insertion depth, a larger volume of the mixture enters the vibration state. As a result, under the same vibration duration, the energy consumed by the mixture per unit time increases, but the increment gradually decreases, showing a trend similar to a quadratic function. The reason is that the acceleration of the vibrator continuously attenuates from the head to the tail of the rod. The smaller vibration acceleration transmitted into the deeper mixture leads to weaker energy input. Therefore, for the same downward insertion distance, the increment of mixture energy consumption per unit time becomes smaller. At the same time, the longer the metal vibrating tube inserted into the mixture, the greater the impedance constraint exerted by the mixture on the tube, which reduces the overall acceleration of the vibrator and further leads to a smaller increment in the energy consumption of the mixture per unit time.

Under different insertion depth conditions, the functional relationship between the mixture energy consumption per unit time and vibration duration is consistent with that between the motor output power and vibration duration. Both exhibit a logarithmic decay trend. Moreover, the smaller the insertion depth, the faster the energy consumption per unit time tends to reach a stable state. This is because when a shorter length of the vibrating tube is inserted into the mixture, the overall acceleration of the vibrator is larger, and a stable paste film is more likely to form rapidly around the casing, allowing both the vibrator vibration and the motor output energy to reach a stable state more quickly.

The impedance of the fresh concrete mixture not only affects the total amount of energy transferred from the internal vibrator to the mixture but also influences the spatial distribution of energy within the mixture. Experimental comparisons show that the vibration energy attenuates rapidly in the mixture in a quasi-exponential form. The decay rate of energy consumption per unit mass and per unit time increases with increasing impedance, while the influence radius of the internal vibrator decreases as the impedance increases.

The mechanism of this phenomenon can be explained as follows. A mixture with higher impedance is more favorable for the transmission of the excitation force of the internal vibrator, forcing the mixture near the vibrating rod to overcome stronger internal medium constraints and thus generating more intense vibration. However, the stronger the vibration of the mixture, the greater the corresponding energy attenuation. Therefore, in a high-impedance mixture, the vibration energy decays more rapidly and more easily drops to the critical intensity required to destroy the initial contact state of the mixture, resulting in a reduction in the influence radius of the internal vibrator.

### 4.3. Vibration Influence Radius and Energy Threshold

#### 4.3.1. Determination of Upper Energy Threshold

The core objective in controlling the upper limit of vibration energy for ternary-graded concrete is to prevent excessive energy input from causing aggregate–paste segregation. The determination of this threshold follows a stepwise logic of “macroscopic performance characterization–microscopic structural quantification–energy value matching,” by which the segregation phenomenon is directly correlated with the energy input.

Taking the D3 ternary-graded concrete with a slump of 180 mm as the test object, a built-in motor eccentric-type internal vibrator was applied for continuous vibration for 55 s at the center of a cylindrical mold. After the concrete had hardened, the segregation region was preliminarily identified using the rebound hammer method. With the contact surface between the vibrator casing and the concrete taken as the origin, the concrete cross-section was divided radially into six testing zones at intervals of 15 mm. In each zone, five measurement points were uniformly selected along the circumferential direction. The surface strength was measured using a rebound hammer, and the average value was calculated.

**Figure 14:**
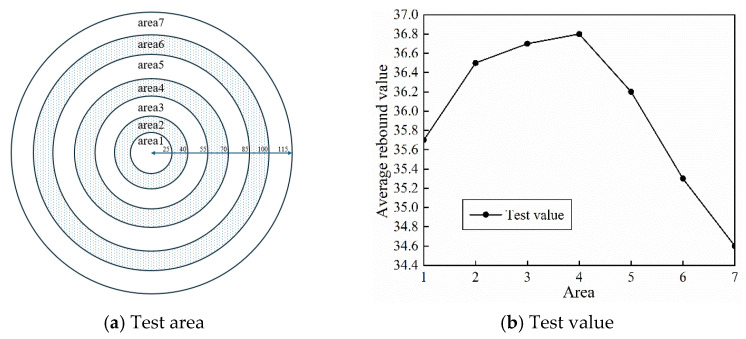
Division of Concrete Vibration Zone and Evaluation of Vibration Quality.

As shown in Figure 14, the average rebound value of Zone I was 35.7, which is 1.0–1.2 lower than that of Zone II (36.5) and Zone III (36.7). In addition, its coefficient of variation reached 0.021, which is significantly higher than the value of 0.008 for the other zones. Based on these results, Zone I was preliminarily identified as the segregation-prone zone, and the segregation boundary was temporarily determined to be 15 mm from the vibration source.

To overcome the limitations of the rebound hammer method, a cross-section image analysis method was further employed to quantitatively determine the segregation boundary. The vibrated concrete specimens were cut radially into prismatic samples with dimensions of 90 mm in length, 50 mm in width, and 200 mm in height. For each test zone, three representative cross-sections were equally selected along the height direction. High-resolution industrial cameras were used to capture images of each section. The images were then processed through grayscale conversion, threshold segmentation, and noise reduction to obtain binary images. Subsequently, the volume fractions of coarse aggregates at the top and bottom of each section were calculated based on pixel statistics and substituted into the segregation evaluation model. The calculation formula of the segregation index (VI) is given in Equation (25).

**Figure 15:**
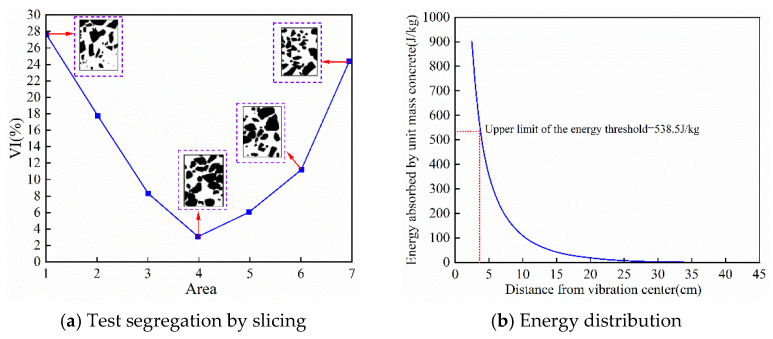
Defining the upper limit of the energy threshold.

The results show that the average (VI) value of Zone I is 27.3 percent, exceeding the critical segregation threshold of 25 percent. The (VI) values of Zones II to VI are all lower than 22 percent and show a decreasing trend with increasing distance from the vibration source. Accordingly, the segregation boundary was accurately revised to 40 mm from the vibration source. Figure 15 illustrates the experimentally determined boundary separating under-compaction and segregation, together with the corresponding vibration energy distribution used to define the upper energy threshold.

(25) $$VI(\%)=2\cdot \frac{\left|V_{at}-V_{ab}\right|}{V_{at}+V_{ab}}\cdot 100\%$$

Finally, based on the energy density attenuation model, the energy value corresponding to the segregation boundary was calculated to determine the upper energy threshold. By substituting the segregation boundary 40 mm, the damping coefficient of the D3 concrete mixture 0.082 N·s/m, and other relevant parameters, the absorbed energy at this position was computed as 538.5 J/kg. An additional 60 s vibration verification test was conducted: when the energy input reached 550 J/kg, the VI value of Zone I increased to 31.2%, indicating aggravated segregation. When the energy was controlled at 538.5 J/kg, the VI value remained stable near the 25% critical limit, with no noticeable expansion of segregation. Thus, the upper energy threshold for vibrating ternary-graded concrete was determined to be 538.5 J/kg.

#### 4.3.2. Determination of Lower Energy Threshold

Using the D3 ternary-graded fresh concrete mixture with a slump of 180 mm as the test object, a built-in motor eccentric-type internal vibrator was applied for 60 s of vibration. After molding and curing 150 × 150 mm specimens, slice samples were prepared at 5 mm intervals. Nuclear Magnetic Resonance (NMR) was first used to measure porosity and locate under-compacted regions. The vibrated concrete specimen was cut radially into rectangular samples measuring 105 mm in length, 50 mm in width, and 350 mm in height. Along the radial direction, the specimen was divided into seven test zones at 15 mm intervals. Each zone was further divided into five parallel segments along the height, and the porosity of each segment was measured.

**Figure 16:**
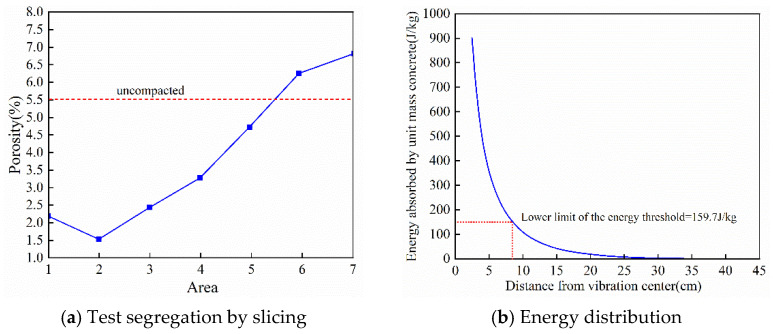
Defining the lower limit of the energy threshold.

The results show that Zone VI (75–90 mm from the vibration source) and Zone VII (90–105 mm) have average total porosities of 5.8% and 6.3%, respectively, both exceeding the 5.5% porosity limit. Zones I to V all exhibit porosities below 5.5%, among which Zone III (30–45 mm) shows the lowest porosity (4.2%). Additionally, the proportion of large pores (>1000 nm) in Zones VI and VII reaches 35%, significantly higher than Zone I’s 18%. Based on these findings, the position 75 mm from the vibration source was preliminarily identified as the critical boundary between compacted and uncompacted regions. Figure 16 illustrates the experimentally determined boundary between under-compaction and adequate compaction, together with the corresponding vibration energy distribution used to define the lower energy threshold.

To verify the porosity-based judgment, compressive strength tests were further carried out. Cylindrical core samples with a diameter of 60 mm and a height of 350 mm were drilled from the compacted region (Zones I–V) and the under-compacted region (Zones VI–VII), with two symmetric cores taken from each zone. A servo-controlled testing system for concrete was used to perform 28-day compressive strength tests at a loading rate of 0.5 MPa/s. The results show that the average compressive strengths of cores from Zones I–V all exceeded the design value of 30 MPa, with Zone III reaching the highest strength (34.2 MPa). In contrast, the average compressive strengths of cores from Zones VI and VII were 27.5 MPa and 25.8 MPa, respectively, both lower than the design value. Their failure mode was brittle fracture, which is clearly different from the plastic failure observed in the compacted region. These findings confirm that the position 75 mm away from the vibration source is the critical boundary between effectively compacted and under-compacted fresh concrete mixture.

Finally, the lower energy threshold was determined by calculating the energy value corresponding to this critical boundary using the energy diffusion model. By substituting the dynamic damping coefficient of the D3 fresh concrete mixture, 0.082 N·s/m, the initial energy density at the vibration source (derived from the energy absorption power of the fresh concrete mixture at a vibration duration of 48 s), and the critical boundary distance of 75 mm, the absorbed energy at this position was obtained as 159.7 J/kg. A verification test with a 45 s vibration duration was additionally conducted: when the input energy was reduced to 150 J/kg, the porosity in Zone V increased to 5.7% and the compressive strength decreased to 29.3 MPa, approaching an under-compacted state; when the energy was controlled at 159.7 J/kg, the porosity in Zone VI remained at the critical value of 5.5% and the strength reached 30.2 MPa, meeting the design requirement. Therefore, the lower energy threshold for vibrating ternary-graded fresh concrete mixture was finally determined to be 159.7 J/kg.

The upper and lower vibration energy thresholds determined in this study were not directly derived from the energy transfer model alone but were obtained through a combined approach integrating experimental identification and energy back-calculation. First, the vibrated concrete specimens were spatially evaluated based on rebound value distribution, cross-sectional image analysis, and porosity measurements, by which the spatial boundary corresponding to the onset of segregation and the critical boundary of insufficient compaction were identified, respectively. This step was conducted purely based on experimental observations to delineate different compaction quality states in space, without invoking any predictions from the energy model.

Subsequently, the measured vibration acceleration responses at these experimentally identified boundary locations were extracted, and the effective average values during the steady vibration stage were taken as representative inputs. These measured accelerations were then substituted into the proposed energy calculation model, together with the known concrete density, dynamic damping coefficient, and natural frequency, to back-calculate the vibration energy absorbed per unit mass of concrete at the corresponding locations. The resulting energy values were defined as the vibration energy thresholds, where the value of 538.5 J·kg^−1^ at 40 mm represents the critical absorbed energy level associated with the onset of segregation, and the value of 159.7 J·kg^−1^ at 75 mm represents the minimum absorbed energy required to just achieve adequate compaction. This approach ensures that the energy thresholds are directly grounded in experimental observations and quantitatively characterized through the energy model.

### 4.4. Experiment-Based Energy Transfer Model for Vibration of Ternary-Graded Fresh Concrete Mixture

Unlike the theoretical expressions in Section 2, the relationships introduced in this section are empirical in nature and are obtained by fitting the experimental data and are, therefore, directly validated through statistical regression.

Based on the experimental results and the energy transfer model proposed in this study, Table 3 summarizes the aggregate grading, recommended vibration time, and safe energy range of three-graded fresh concrete under typical slump conditions and further identifies the corresponding effective vibration influence range.

The results of the vibration tests show that, for three-graded concrete subjected to vibration, the dynamic damping increases concurrently with the aggregate-specific surface area and the progression of densification. According to the experimental regression results, the damping can be formulated as a linear combination of the initial damping, the specific surface area contribution, and the densification rate term, i.e.,

(26) $$\zeta =\zeta_{0}+\alpha S+\beta \phi ’,$$

where *ζ* is the total damping, *ζ*_0_ is the initial damping, *S* is the specific surface area of aggregates, *φ’* is the rate of change in concrete compactness, and *α* and *β* are the response coefficients.

The vibration tests indicate that the energy absorbed per unit mass of concrete decreases with increasing damping and exhibits a decaying trend with vibration time. Based on the experimental fitting results, the unit mass energy absorption is formulated as an inverse function of damping, with an exponential time-decay term incorporated to characterize the evolution of energy absorption capacity. Accordingly, the unit mass energy absorption model for three-graded concrete is expressed as

(27) $$E_{t}=\frac{E_{0}}{1+\zeta }e^{-\gamma t},$$

where *E_t_* is the energy absorption per unit mass, *E*_0_ is the initial energy, *ζ* is the damping, *γ* is the attenuation coefficient, and *t* is the vibration time.

Experimental results indicate a strong nonlinear dependence between the excitation energy absorbed by the concrete and the acceleration of the vibrating poker. On the basis of the measured acceleration and energy absorption fitting results, the absorbed energy of the fresh concrete is represented as an exponential function of the poker acceleration, thereby capturing the amplification effect of excitation intensity. The model can be written as

(28) $$E_{a}=ka{\left(t\right)}^{p},$$

where *E_a_* is the energy absorbed by the mixture induced by the casing vibration, *k* is a comprehensive coefficient, *a*(*t*) is the vibrator acceleration, and *p* is the response exponent.

The vibration test results indicate that the energy absorption of concrete exhibits a decay characteristic of “rapid initially followed by gradual attenuation” with increasing vibration time. Based on the fitted time-history results, a logarithmic decay function is adopted to describe the evolution of the energy absorption capacity, which can be expressed as

(29) $$E_{\ln}=A-B\ln\left(t\right),$$

where *A* and *B* are fitting coefficients, and *t* is the vibration time.

The experimental results indicate that the energy absorption of concrete increases with the insertion depth of the vibrating poker, while exhibiting an overall nonlinear trend. Based on the fitted relationship between insertion depth and energy absorption, a quadratic function is adopted to describe the effect of insertion depth on energy absorption, which can be expressed as

(30) $$E_{z}=a_{1}z^{2}+b_{1}z+c_{1},$$

where *z* is the insertion depth of the internal vibrator, and *a*_1_, *b*_1_ and *c*_1_ are fitting coefficients.

The vibration tests indicate that the excitation energy within the concrete exhibits a rapid attenuation with increasing spatial distance. Based on the fitted spatial distribution results, an exponential function is adopted to describe the distance-dependent decay of energy, and the corresponding model can be expressed as

(31) $$E_{r}=E_{0}e^{-\lambda r^{q}},$$

where *r* is the distance from the vibration center of the fresh concrete mixture, *λ* is the attenuation coefficient, and q is the diffusion exponent.

Based on the vibration test results, the energy absorption behavior of three-graded concrete during vibration can be decomposed into several mutually independent yet simultaneously acting control processes. Analysis of the measured acceleration, absorbed energy power, and spatial attenuation data indicates that the vibrator casing acceleration *a*(*t*) governs the instantaneous energy input intensity, while the equivalent damping of the concrete can be jointly characterized by the initial damping, the aggregate-specific surface area, and the rate of densification. The energy absorption capacity exhibits a logarithmic or exponential decay with vibration time. Meanwhile, the insertion depth of the vibrator controls the extent of vertical energy participation, and the spatial diffusion function determines the radial attenuation of energy. Based on these experimentally identified governing variables, each influencing term was independently fitted and subsequently coupled in a multiplicative form, resulting in a comprehensive empirical model that describes the transmission and attenuation of vibration energy across the coupled time–depth–space dimensions. All model parameters were obtained through experimental inversion, enabling a quantitative representation of the realistic vibration response of three-graded concrete. The final expression of the model is given as follows:

(32) $$E\left(t,z,r\right)=\frac{ka{\left(t\right)}^{p}}{1+\zeta_{0}+\alpha S+\beta \phi ’}\left[A-B\ln\left(t\right)\right]E_{0}e^{-\lambda r^{q}}\left(a_{1}z^{2}+b_{1}z+c_{1}\right).$$

To further examine the external rationality of the proposed model, a comparison was conducted with the vibration energy transfer model and experimental results reported by Li et al. (2023) [42], who investigated the energy distribution of fresh concrete subjected to an internal vibrator with a built-in motor.

Although the two studies differ in model assumptions and parameter definitions, both frameworks describe the spatial attenuation of vibration energy in concrete using exponential-type decay functions. The predicted energy density in the present study exhibits a rapid attenuation with increasing distance from the vibration source, which is consistent with the attenuation trend reported by Li et al. (2023) [42].

In addition, the influence radius estimated by the present model is of the same order of magnitude as that obtained from the published experimental results, indicating that the proposed framework captures the essential characteristics of vibration energy propagation in fresh concrete. These consistencies support the external rationality and general applicability of the proposed model, despite differences in experimental configurations and concrete compositions.

It should be noted that the proposed model is established based on a specific aggregate gradation, a limited range of vibration frequencies, and a cement-based binder system. The applicability of the model to other aggregate types or agglomerate structures, different vibration parameters, and alternative binder systems has not been fully examined and requires further investigation through future experimental and field studies.

## 5. Conclusions

To evaluate the vibration quality of ternary-graded fresh concrete mixture, a vibration energy transfer model applicable to ternary-graded concrete was established based on the working mechanism of the internal vibrator and vibration energy theory. The effects of concrete flow damping, paste–aggregate ratio, specific surface area of coarse aggregates, and natural frequency on energy transfer characteristics were systematically analyzed. Based on this model, the spatiotemporal distribution of internal energy in concrete under different combinations of vibration process parameters was simulated.

During the study, acceleration and energy data obtained from the settling-ball tests and measured acceleration responses were used. Ternary-graded concrete was idealized as a homogeneous medium, and the vibrator casing was modeled as a vibration system constrained by elastic springs and non-viscous damping. On this basis, an empirical model for the energy absorption of concrete during vibration was inversely identified, and the applicability and accuracy of the model were verified.

The experimental results indicate that poorer concrete flowability and a higher proportion of coarse aggregates lead to a faster energy attenuation rate in ternary-graded fresh concrete mixture. However, under these conditions, the concrete exhibits a stronger capacity to absorb vibration energy, resulting in a corresponding increase in the energy transfer efficiency of the vibrator. With increasing single vibration duration, both the absorbed energy and the energy transfer efficiency gradually decrease and then tend to stabilize. The overall system energy transfer efficiency remains higher than 55 percent on average and can reach a maximum of 73.5 percent.

The proposed vibration energy transfer model and vibration quality evaluation method can be integrated into digital vibration equipment and intelligent construction systems, enabling real-time evaluation and precise control of the vibration quality of ternary-graded fresh concrete mixture. The method shows good applicability and strong engineering promotion value for vibration construction under vertical insertion conditions using internal vibrators.

Specifically, for three-graded fresh concrete with slump values ranging from approximately 60 to 180 mm, effective vibration can be achieved by controlling the vibration duration within 30–65 s, maintaining the absorbed energy within the safe range of 159.7–538.5 J·kg^−1^, and limiting the effective vibration influence radius to 22–85 mm, as summarized in Table 3, thereby avoiding both insufficient compaction and vibration-induced segregation.

In conclusion, this study establishes an energy-based framework for vibration compaction in three-graded fresh concrete, providing a quantitative link between vibration parameters, energy transfer, and compaction quality. By introducing measurable energy indicators and defining safe energy ranges, the proposed approach contributes to improving current vibration practices from experience-based operation toward more standardized and performance-oriented vibration criteria. These findings are particularly relevant for mass concrete construction, including dams and hydraulic infrastructures, where effective vibration control is critical to ensuring structural integrity and long-term durability. The proposed framework offers a rational basis for selecting vibration parameters and controlling vibration influence ranges in large-volume concrete placements.

Future research may further extend this framework toward intelligent vibration control strategies, such as AI-based real-time adjustment of vibration parameters, and explore its applicability to emerging binder systems, including geopolymer and low-carbon concretes, to support sustainable development in hydraulic engineering.

## Figures and Tables

**Table 1:** Parameters of Internal Vibrators in Air.

Parameters	Characters	Unit	Value
Output power of the motor	P_o_	W	275
Vibration acceleration of the housing	a_o_	m/s^2^	653
Vibration frequency of the housing	f_o_	Hz	192
Radius of the housing	r	cm	3.5
Length of the housing	H	cm	42
Eccentric mass	m_e_	g	195
Eccentric distance	e	mm	12.5
Rotating speed of the eccentric rod	N	rpm	11,468
Outer radius of the bearing inner rings	r_b_	cm	1.2
Friction coefficient of the bearings	μ	/	0.0015

**Table 2:** Concrete Sample Mix Proportion.

Series	SL (mm)	Unit Mass (kg/m^3^)								
		Coarse Aggregate Particle Size Grading (mm)				Fine Aggregate	Cement	Water	Admixture	Water-Reducing Agent
		5–20	20–40	40–80	Grading Ratio					
C1	67	714	714	0	5:5:0	784	187	123	114	4.5
C2	121	714	714	0	5:5:0	784	187	123	114	4.7
C3	181	714	714	0	5:5:0	784	187	123	114	4.9
C4	120	571	857	0	4:6:0	784	187	123	114	4.7
C5	121	857	571	0	6:4:0	784	187	123	114	4.7
D1	67	428	428	572	3:3:4	784	187	123	114	4.5
D2	121	428	428	572	3:3:4	784	187	123	114	4.7
D3	181	428	428	572	3:3:4	784	187	123	114	4.9
D4	120	428	572	428	3:4:3	784	187	123	114	4.7
D5	123	572	428	428	4:3:3	784	187	123	114	4.7

**Table 3:** Summary Table of Vibration Parameters and Safety Energy of Three Graded Freshly Mixed Concrete.

No.	Slump Range SL/mm	Aggregate Gradation	Recommended Vibration Time/s	Safe Energy Range/(J·kg^−1^)	Effective Vibration Influence Radius/mm
D1	67	Triple grading	45–65	159.7–538.5	22–55
D2	121	Triple grading	35–60	159.7–538.5	35–75
D3	181	Triple grading	30–60	159.7–538.5	40–85
D4	120	Triple grading	35–60	159.7–538.5	35–75
D5	123	Triple grading	35–60	159.7–538.5	35–75

## Data Availability

The original contributions presented in this study are included in the article. Further inquiries can be directed to the corresponding author.
